# Cost-Effectiveness Analysis of Herpes Zoster Vaccination in a Chinese Population: Recombinant Subunit Vaccine versus Live Attenuated Vaccine

**DOI:** 10.3390/vaccines12080872

**Published:** 2024-08-01

**Authors:** Jiaqi Wang, Pengfei Jin, Hui Jin, Qiang Wang, Fengcai Zhu, Jingxin Li

**Affiliations:** 1 Department of Epidemiology and Health Statistics, School of Public Health, Southeast University, 87 Dingjiaqiao Avenue, Nanjing 210009, China; wangjqseu@163.com (J.W.); jinhui_hld@163.com (H.J.); 2 Jiangsu Provincial Medical Innovation Center, National Health Commission Key Laboratory of Enteric Pathogenic Microbiology, Jiangsu Provincial Center for Disease Control and Prevention, Nanjing 210009, China; kingpfph@sina.com; 3 Key Laboratory of Environmental Medicine Engineering, Ministry of Education, School of Public Health, Southeast University, Nanjing 210009, China; 4 Department of Epidemiology, School of Public Health, Fudan University, Shanghai 200433, China; wangqiangepi@163.com

**Keywords:** herpes zoster vaccine, cost-effectiveness analysis, recombinant subunit vaccine, live attenuated vaccine

## Abstract

Background: Currently, the recombinant subunit vaccine and live attenuated vaccine in the prevention of herpes zoster are approved for marketing in China. This study aims to evaluate the cost-effectiveness of the recombinant subunit vaccine and the live attenuated vaccine in the Chinese population. Methods: A decision tree–Markov analysis model was utilized to estimate expected costs and quality-adjusted life years (QALYs), comparing the lifetime cost-effectiveness of vaccination with the recombinant subunit vaccine (London, United Kingdom, Shingrix, GSK) to that of the live attenuated vaccine (Changchun, China, Ganwei, Changchun Bcht) in the Chinese population, with the primary outcome measure being the incremental cost-effectiveness ratio (ICER). Results: In the base-case analysis, the ICERs for the recombinant subunit vaccine ranged by age from USD 3428 to USD 5743 per QALY, while the ICERs for the live attenuated vaccine ranged from USD 4017 to USD 18,254 per QALY, compared with no vaccination. Among all age groups, the category of 60 to 69 years was the optimal age for vaccination. The results were most sensitive to changes in herpes zoster incidence, vaccine efficacy, and discount rate. Even with a two-dose compliance rate of 20% for the recombinant subunit vaccine, vaccination remained cost-effective. ZVL would need to reduce costs by at least 12.2% compared to RZV to have a cost-effectiveness advantage. Conclusions: The recombinant subunit vaccine and the live attenuated vaccine were both cost-effective in the Chinese population, but, relatively, the recombinant subunit vaccine had a greater advantage in disease prevention and cost-effectiveness in all age groups above 50 years.

## 1. Introduction

Herpes zoster (HZ) is a viral infectious disease caused by the varicella-zoster virus (VZV) [1], characterized by a vesicular rash that is generally limited to a single dermatome and unilateral radicular pain [2,3]. The virus remains dormant in nerve ganglia after infecting the body and can reactivate when the immune system is weakened, triggering shingles [3,4]. The primary complications of shingles include postherpetic neuralgia (PHN), ocular involvement, neurological issues, motor nerve damage, and more severe disseminated diseases in immunocompromised patients [2]. The most common complication is PHN, with pain lasting for months to even years, affecting approximately 20% of adults and being more prevalent in the elderly [5], significantly impacting patients’ quality of life [6].

HZ is highly prevalent worldwide, with an increasing incidence annually [7,8]. Approximately 95% of adults are latently infected with the varicella-zoster virus, with about one-third experiencing HZ outbreaks [2]. The recurrence risk of HZ is between 1% and 6% [8], increasing with age and imposing a significant burden on healthcare systems and economies globally [9]. In China, the incidence of HZ is gradually rising due to population aging and the increase in chronic diseases [10]. China’s total population exceeds 1.4 billion. In 2020, it was estimated that residents aged 50 years or older accounted for approximately 35% of the Chinese population [11]. According to statistics from the Chinese Center for Disease Control and Prevention, there are hundreds of thousands of HZ cases in China every year [12], and its increasing trend has caused a considerable burden on China’s healthcare system and economy.

Vaccination is effective in controlling HZ and its related complications [2,13,14]. In China, the available vaccines for preventing HZ are GSK’s recombinant subunit vaccine and Changchun Bcht’s live attenuated vaccine, launched in 2019 and 2023, respectively. The protective efficacy of the recombinant subunit vaccine (London, United Kingdom, Shingrix, GSK) against HZ remains above 90% in all age groups over 50 years. However, compared to the live attenuated vaccine, the recombinant subunit vaccine requires two doses and has a relatively high incidence and severity of adverse reactions after vaccination [14,15]. In contrast, the domestic live attenuated vaccine (Changchun, China, Ganwei, Changchun Bcht) provides about 57% protection against HZ, which is more affordable and requires only one dose [16]. In China, the herpes zoster vaccine is currently a non-immunization program vaccine, and recipients need to pay for it themselves. With both the recombinant subunit vaccine and the live attenuated vaccine gradually being introduced in China, there is an urgent need for health policy makers to address the issue of how to maximize benefits at the lowest cost within limited resources, including the selection of immunized populations and the cost of willingness to pay for the vaccine.

Vaccine economic evaluation plays a crucial role in health decision-making, especially in resource-limited settings, and it helps to identify optimal immunization programs through cost-effectiveness analysis. Studies in Europe and the United States have shown that the live attenuated vaccine (Zostavax, Merck) is generally cost-effective [17], but cost-effectiveness varies widely between studies due to differences in vaccine price, vaccine efficacy, protection durability, prevalence characteristics, and economic parameters. Shingrix was more cost-effective than Zostavax for the same vaccine price [18,19,20]. However, it is not yet known whether the live attenuated vaccine (Ganwei, Changchun Bcht) and Shingrix are cost-effective at China’s economic level and herpes zoster prevalence. In this study, we compare the cost-effectiveness of the recombinant subunit vaccine and live attenuated vaccine and provide scientific and rational recommendations for the vaccination strategy of HZ vaccines for the vast immunization target population in China, which is important for the development of vaccine policies and the improvement of public vaccination rates.

## 2. Methods

### 2.1. Model Structure

**Figure 1:**
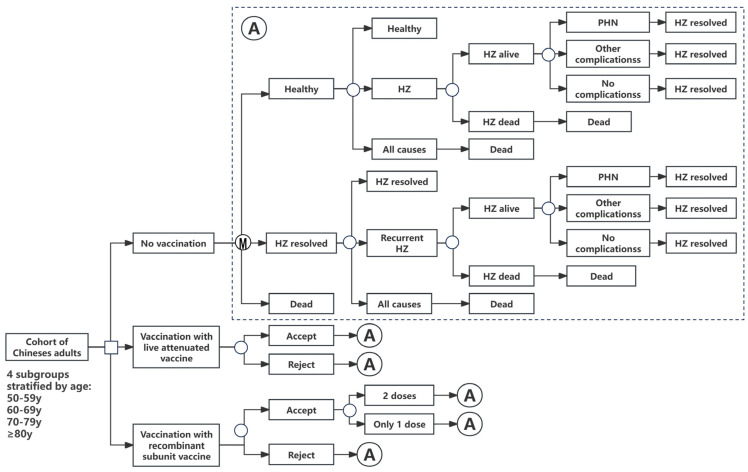
Simplified decision–analytic model. M = Markov node.

In TreeAge Pro 2022 (version R1.2), a decision tree–Markov model is developed to predict the costs and health outcomes of three strategies: no vaccination, vaccination with the live attenuated vaccine, and vaccination with the recombinant subunit vaccine (Figure 1). A cohort of immunocompetent Chinese adults aged 50 years or older, stratified by age group (50–59, 60–69, 70–79, and ≥80 years), was simulated, and all simulated populations entered the model in good health starting at the age of vaccination for each age group and were cycled through the model annually over the course of a 40-year life cycle. Each year of the simulation, an individual may experience HZ, PHN, other complications, or death, with the model incorporating the possibility of HZ recurrence.

Square nodes represent decisions, while circular nodes represent chance events. “HZ resolved” indicates a health state after an episode of HZ in which the person has recovered from HZ, PHN, or other complications. HZ stands for herpes zoster; PHN stands for postherpetic neuralgia.

### 2.2. Transition Probabilities

We obtained transition probabilities, costs, and other model inputs from the published literature. A literature search was conducted on PubMed and Web of Science from January 2000 to December 2023. This search aimed to obtain data specific to the Chinese population; if such data were unavailable, data for the Asian population were chosen, trying to select studies with larger sample sizes and more recent dates. Ethical approval is not required because the model inputs are derived from the literature.

Age-specific HZ incidence rate data are derived from a meta-analysis of 12 studies involving nearly 26 million people in China [9]; the conditional probabilities of PHN and complications such as eye, ear, nervous system, and other conditions during HZ are determined based on large-scale clinical data collected from various medical institutions in the city of Yichang from 1 January 2016 to 31 December 2017 [21]; the mortality rate for HZ is adopted from a study in Japan due to similar disease models [22]; the recurrence rate of HZ is based on the medical record data of 218,683 individuals aged 50 years or older in the city of Ningbo from 1 January 2015 to 31 December 2017 [23]; and age-adjusted all-cause mortality rates are also included in the model, with data up to 1 November 2022, sourced from the National Bureau of Statistics of China (Table S1) [11].

Parameters related to the vaccine can be found in Table S1. Recent survey results from a systematic review and meta-analysis of the Chinese population show a 35% willingness to vaccinate against HZ [24]. Considering the contraindication of the live attenuated vaccine in individuals with low immune function, we set the vaccine coverage rate in the analysis to 30% of the total population. Assuming all recombinant subunit vaccine recipients receive two doses of the vaccine within two months, the effectiveness data of the recombinant subunit vaccine over ten years is derived from four randomized controlled trials [25,26,27,28]. Based on the Phase III clinical trial report of the domestic live attenuated vaccine, the efficacy of the domestic live attenuated vaccine is similar to the efficacy data of the live attenuated vaccine from Merck in the United States. Hence, the simulation is based on Merck’s live attenuated vaccine follow-up data from up to 11 years [16,29,30,31,32]. The vaccine efficacy models of the recombinant subunit vaccine and the live attenuated vaccine against HZ over time are based on linear functions extrapolated from published data.

### 2.3. Costs and Quality of Life

The associated costs and quality-adjusted life years (QALYs) for each HZ are displayed in Table S2. Disease costs comprise direct and indirect costs. Direct costs include direct medical costs and non-medical costs, all adjusted to 2023 national prices using the Chinese Consumer Price Index. Vaccine prices in this study are derived from the disease prevention and control center at the clinical trial site (USD 453.55 per dose for the recombinant subunit vaccine and USD 194.28 per dose for the live attenuated vaccine), whereas the disease costs and management costs (USD 4.24 per dose) are obtained from the literature [33]. Direct medical costs for complications like postherpetic neuralgia (PHN) come from data reported to the National Health Statistics Information Network by 372 hospitals in the Hunan province from 2017 to 2019, as well as outpatient electronic medical records from Ningbo [23,34]. Direct non-medical costs and indirect costs are based on hospital information systems in the province of Jiangsu from 2020 to 2021, estimating economic losses due to patients or their caregivers being unable to work due to illness [35]. All costs are reported in USD adjusted to 2023 (1 USD = 7.0467 CNY) and discounted at an annual rate of 5% [36,37]. QALYs are a vital measure of health benefits. Age- and gender-specific utility scores in different health states are sourced from national health service survey data [38,39]. Utility values for complications like HZ and PHN are obtained from hospitals’ EQ-5D surveys (HZ’s QALY is 0.85, PHN’s QALY is 0.74) [40].

### 2.4. Cost-Effectiveness Analysis and Secondary Outcomes

Cost-effectiveness analysis is performed from a societal perspective, which can help to provide a more complete assessment of the cost-effectiveness of immunization programs. The leading indicator involves calculating the incremental cost-effectiveness ratio (ICER) of two vaccines across different age groups, measured in terms of cost per QALY. An annual discount rate of 5% was used [37], indicating the rate at which costs and outcomes are discounted to account for time preference. According to the World Health Organization’s (WHO) recommendations on the economic evaluation of vaccines, “a health intervention should be considered cost-effective if the ICER is between one and three times the GDP per capita of that country” [41]. In 2023, for example, China’s GDP per capita will be USD 12,681 [42]. Accordingly, the willingness to pay (WTP) threshold for vaccines is set at one time the GDP per capita. Secondary outcomes include the number of HZ cases, PHN cases, and total costs.

### 2.5. Sensitivity Analysis

We performed a one-way sensitivity analysis on the parameters, where the model inputs were varied individually between their lower and upper limits while keeping other values at their base-case levels to identify the key parameters affecting model stability. Furthermore, within the ranges of parameter values, we applied a Monte Carlo random model 10,000 times for probabilistic sensitivity analysis. We generated a cost-effectiveness acceptability curve to compare the probabilities of three vaccination strategies (vaccination with two doses of recombinant subunit vaccine, vaccination with one dose of live attenuated vaccine, and no vaccination) becoming the preferred strategies. We also performed a series of scenario analyses to explore the impact of varying compliance rates for receiving two doses of the recombinant subunit vaccine (ranging from 20% to 100%) and performed a threshold analysis on the cost of the live attenuated vaccine to comprehensively assess the uncertainty in the model.

## 3. Results

### 3.1. Base-Case Analysis

Compared with no vaccination, vaccination with the recombinant subunit vaccine can prevent 43% of HZ cases and 47% of PHN in the 50–59 age group, as well as 80% of HZ cases and 95% of PHN in the 80 and older age group over a lifetime; vaccination with live attenuated vaccine can prevent 10% of HZ cases and 8% of PHN in the 50–59 age group over a lifetime, and 17% of HZ cases in the 70–79 age group and 8% of PHN in the 80 and older age group over a lifetime. Vaccination with the live attenuated vaccine prevents 10% of HZ cases, and 20% of PHN in the 80 and older age group. Vaccination with the recombinant subunit vaccine prevents more cases of HZ and its complications than vaccination with the live attenuated vaccine in all age groups over 50 years (Table 1).

In the age group 50 years and older, the incremental expected cost and expected QALY of receiving the recombinant subunit vaccine are higher than those of receiving the live attenuated vaccine compared with no vaccination. In the 80 and older age group, the largest cost difference is USD 56,226 higher for receiving the live attenuated vaccine compared with no vaccination, while the cost of receiving the recombinant subunit vaccine is USD 117,262 higher than receiving the live attenuated vaccine. In the 60–69 age group, vaccination with the live attenuated vaccine results in the greatest number of QALYs compared with no vaccination, 12.10 QALYs, while vaccination with the recombinant subunit vaccine results in 31.72 QALYs. Vaccination with the recombinant subunit vaccine is cost-effective compared with no vaccination for all age groups (ICERs are all below the WTP threshold of USD 12,681 per QALY gained) live attenuated vaccination is cost-effective in all age groups except the 80+ age group, and recombinant subunit and live attenuated vaccines are most cost-effective in the 60–69 age group.

### 3.2. One-Way Sensitivity Analyses

**Figure 2:**
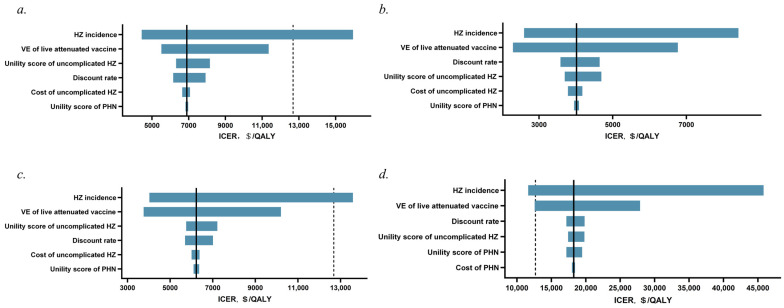
One-way sensitivity analysis of live attenuated vaccine versus no vaccination. (**a**) 50−59 years, (**b**) 60−69 years, (**c**) 70−79 years, (**d**) ≥80 years. The dotted vertical line indicates one time the per capita GDP of USD 12,681/QALY. HZ = herpes zoster; PHN = postherpetic neuralgia; VE = vaccine efficacy.

All model input data were assessed in a univariate sensitivity analysis. The top six factors influencing the ICER of vaccination with the live attenuated vaccine compared with non-vaccination across different age groups are illustrated in tornado diagrams (Figure 2a–d). In general, the results are most sensitive to the incidence of HZ and vaccine efficacy, while they are less sensitive to PHN’s incidence, costs, and utility values.

**Figure 3:**
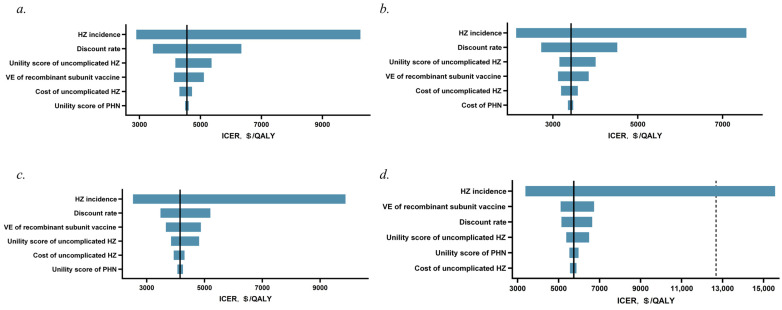
One-way sensitivity analysis of recombinant subunit vaccine versus no vaccination. (**a**) 50−59 years, (**b**) 60−69 years, (**c**) 70−79 years, (**d**) ≥80 years. The dotted vertical line indicates one time the per capita GDP of USD 12,681/QALY. HZ = herpes zoster; PHN = postherpetic neuralgia; VE = vaccine efficacy.

The six key factors influencing the ICER of vaccination with the recombinant subunit vaccine compared with non-vaccination in each age group are shown in the tornado diagrams (Figure 3a–d). Overall, the results are most sensitive to the incidence and discount rate of HZ, while they are less sensitive to the incidence, cost, and utility values of PHN.

### 3.3. Probabilistic Sensitivity Analyses

**Figure 4:**
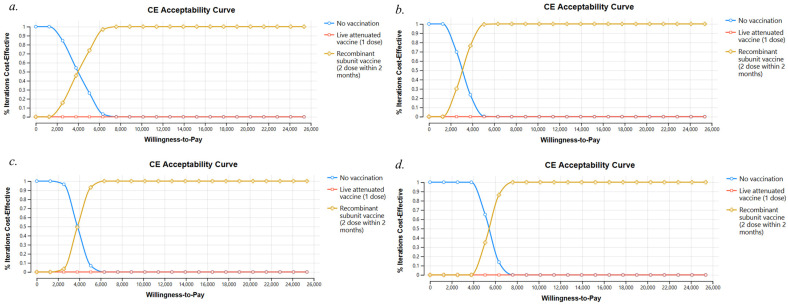
Cost-effectiveness acceptance curves for different age groups. (**a**) 50−59 years, (**b**) 60−69 years, (**c**) 70−79 years, (**d**) ≥80 years.

In a probabilistic sensitivity analysis with 10,000 simulations, a willingness-to-pay threshold of USD 12,681 per QALY was identified. Across all age groups, the proportion of recombinant subunit vaccination strategy, which has become the preferred strategy in all age groups, is close to 100%. In contrast, the live attenuated vaccination strategy was almost completely dominated by 0% (Figure 4a–d).

**Figure 5:**
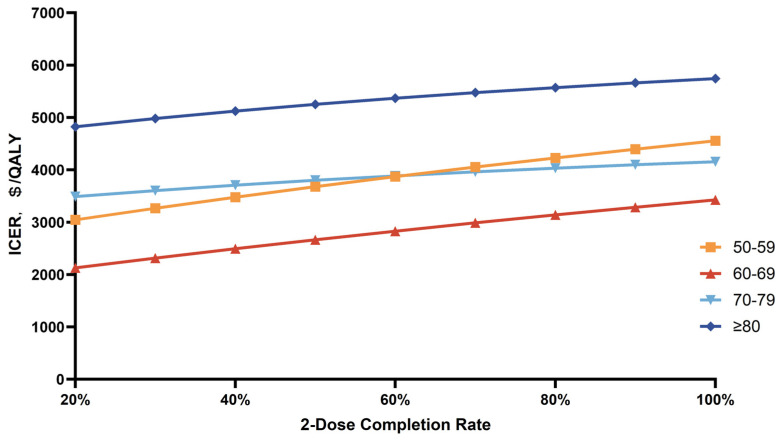
ICERs for scenario analysis for vaccination with recombinant subunit vaccine compared with no vaccination.

In the scenario analysis, the impact of different compliance rates for completing two doses of recombinant subunit vaccine on ICER varies by age (Figure 5). A lower completion rate of two doses of recombinant subunit vaccine is associated with a decrease in ICER. In a scenario where the compliance rate for two doses is 20%, the vaccination with recombinant subunit vaccine strategy is cost-effective for all age groups.

Further exploration of the impact of vaccine costs on the cost–effectiveness of live attenuated vaccines compared with recombinant subunit vaccines reveals that in the age groups of 50–59 years, 60–69 years, and 70–79 years, the cost of the live attenuated vaccine decreases to USD 134.4 (a 30.8% cost reduction), USD 170.5 (a 12.2% cost reduction), and USD 136.7 (a 29.6% cost reduction), respectively, making live attenuated vaccine cost–effective.

## 4. Discussion

This study aims to conduct a comparative analysis for the first time from a societal perspective on the health and economic effects of vaccinating the population aged 50 years or older in China with the live attenuated vaccine and the recombinant subunit vaccine. Recombinant subunit vaccines were found to be more advantageous in terms of disease prevention and cost-effectiveness for all age groups over 50 years. The recombinant subunit vaccine was cost-effective in all age groups over 50 years compared with no vaccination, and with a two-dose compliance rate of 20% for the recombinant subunit vaccine, vaccination was still cost-effective; for the live attenuated vaccine, it was cost-effective in all age groups except the 80+ group; and the optimal age for both the recombinant subunit vaccine and the live attenuated vaccine was 60–69 years. In our study, it was observed that among individuals aged 50 years or older, the economic attractiveness of receiving the recombinant subunit vaccine was higher, despite its high cost being a major influencing factor for HZ vaccine uptake [43]. To increase vaccination rates and reduce the burden of the disease, vaccination with the live attenuated vaccine is also an alternative, but it may require a price reduction of at least 12.2%.

A search of PubMed up to March 2024 found that other researchers have explored the cost-effectiveness of the recombinant subunit vaccine and the live attenuated vaccine in other countries, such as the United States, Canada, the Netherlands, and Belgium, and have consistently shown that the use of the recombinant subunit vaccine is more cost-effective than the live attenuated vaccine across all age groups, with more favorable results for adults aged 60 and above, which aligns with the findings of our study [18,19,44,45,46,47]. In the study conducted in the United States, vaccination with the live attenuated vaccine is also cost-effective, considering the lower cost of the domestically produced live attenuated vaccine; the cost of the live attenuated vaccine is $223. Additionally, based on a recent prospective cohort study covering nearly 9.5 million person-years, the protective effect of the live attenuated vaccine against HZ after ten years is 38.1%, and against PHN after ten years it is 59.0% [48], which is higher than the results of clinical trials. Therefore, vaccination with the live attenuated vaccine may be cost-effective compared with not vaccinating, as indicated by 67% of the 11 studies conducted after 2014 [17].

In our study, the cost-effectiveness of the live attenuated vaccine is primarily influenced by the incidence and efficacy of HZ. The incidence rate is a core indicator of the disease burden, which directly determines the overall magnitude and severity of the disease; secondly, there may be uncertainty in the incidence rate; the actual incidence rate is often higher than the reported incidence rate due to the limitations of the disease surveillance and reporting system. Furthermore, the incidence rate can change over time with improvements in healthcare conditions. Therefore, the incidence rate is generally one of our most important and influential parameters. For live attenuated vaccines, cost-effectiveness decreases as the incidence of HZ increases, and ICERs exceed the WTP threshold for those aged 80 years and older, which may be largely due to the lower efficacy of live attenuated vaccines. Finally, vaccine efficacy and waning efficacy determine changes in HZ incidence over time, thus also affecting cost-effectiveness outcomes. In addition, the recombinant subunit vaccine is primarily affected by the HZ incidence rate and discount rate. This is consistent with previous research findings [17,49,50,51]. The choice of discount rate has a strong influence on the weighting of the distant effects, and the health effects of vaccination are often reflected in the more distant future, so the cost-effectiveness outcomes of vaccination are quite sensitive to the discount rate.

The limitations of this study are mainly due to the limited availability of model parameters. These parameters mainly include the long-term efficacy of the vaccine. Since the efficacy of the domestic live attenuated vaccine in Phase III clinical trials was 62.7%, 64.4%, and 18.6% in the age groups of 50–59, 60–69, and 70 years and older, respectively, which was similar to the efficacy of the Merck live attenuated vaccine of 69.8%, 63.9%, and 37.6% [30,52], the long-term efficacy data for the Merck live attenuated vaccine were used to model the long-term efficacy data for the domestic live attenuated vaccine in our study. Moreover, the indication for the domestic live attenuated vaccine is for people aged 40 years and older, but data are lacking on the incidence of HZ in younger age groups. In addition, the study did not separately consider the impact of quality-adjusted life years for HZ and other complications (e.g., ocular complications). Finally, the particular source of the data may have an impact on the results; for example, the use of hospital-based morbidity data may underestimate actual morbidity by omitting unseen patients, thus overestimating cost-effectiveness, while the use of rural area-based burden-of-disease costs may underestimate cost-effectiveness because of the relative scarcity of healthcare resources and lower prices for healthcare services in rural areas.

With the introduction of a domestically produced live attenuated vaccine, Chinese residents now have an additional option to prevent HZ and its complications through vaccination. Although the recombinant subunit vaccine shows some advantages in cost-effectiveness analyses, the live attenuated vaccine also has a place in public health strategies due to its wide age range and easy vaccination procedures. From a policymaking perspective, the promotion of the live attenuated vaccine may have a positive impact on improving access, convenience, and adherence to vaccination. According to publicly available data from the WHO, there are currently 54 herpes zoster vaccines in the world, 15 of which are in clinical development [53]. In the future, as other vaccines become available and more data are accumulated, the vaccination strategy for the herpes zoster vaccine in the Chinese population will become more complex, and its health economic evaluation will face greater challenges.

## 5. Conclusions

Vaccination with the domestically produced live attenuated vaccine and the recombinant subunit vaccine showed significant cost-effectiveness compared with no vaccination. However, the recombinant subunit vaccine showed greater benefits in terms of disease prevention and cost-effectiveness. In particular, 60–69 years was identified as the optimal age for vaccination. This finding provides an important basis for the optimization of vaccination strategies in China and helps to maximize health benefits and economic benefits in public health policy formulation.

## Figures and Tables

**Table 1:** From a societal perspective, the classification results of receiving live attenuated vaccine, receiving recombinant subunit vaccine, and not receiving any vaccine in the base case, along with ICER (by age group) per thousand population, are displayed.

Vaccination Strategy	Cases, n	PHN Cases, n	Cost, USD	QALYs, n	Incremental Costs, USD	Incremental QALYs, n	ICER, USD/QALY
50 years							
No vaccination	287	62	120,439	14,119	-	-	-
Live attenuated vaccine	257	57	173,280	14,127	52,841	7.66	6900
Recombinant subunit vaccine	164	33	235,419	14,145	114,980	25.24	4555
60 years							
No vaccination	240	57	130,046	11,467	-	-	-
Live attenuated vaccine	201	47	178,670	11,479	48,624	12.10	4017
Recombinant subunit vaccine	102	17	238,768	11,498	108,723	31.72	3428
70 years							
No vaccination	171	46	103,896	8456	-	-	-
Live attenuated vaccine	142	38	155,782	8464	51,886	8.33	6230
Recombinant subunit vaccine	50	6	216,231	8483	112,335	27.02	4157
80 years							
No vaccination	114	40	77,978	5297	-	-	-
Live attenuated vaccine	102	32	134,204	5301	56,226	3.08	18,254
Recombinant subunit vaccine	23	2	195,240	5318	117,262	20.42	5743

PHN = postherpetic neuralgia; ICER = incremental cost-effectiveness ratio; QALY = quality-adjusted life-year.

## Data Availability

The data presented in this study are available upon request from the corresponding author.

## Supplementary Materials

The following supporting information can be downloaded at: https://www.mdpi.com/article/10.3390/vaccines12080872/s1, Table S1: Probability inputs for the analysis; Table S2: Costs and quality-of-life inputs for the analysis.
