# Supplementary material for: Cost-Effectiveness Analysis of Herpes Zoster Vaccination in a Chinese Population: Recombinant Subunit Vaccine versus Live Attenuated Vaccine
Source: Vaccines (Basel). 2024 Aug 1;12(8):872. doi: 10.3390/vaccines12080872 (PMC11359938; doi:10.3390/vaccines12080872)
Supplement: Supplementary file 1 [file vaccines-12-00872-s001.zip › vaccines-3078006-supplementary/vaccines-3078006-supplementary/Supplementary File.docx]

**Table S1** Probability inputs for the analysis [1-16].

| Variable | Base case value | Range | Distribution | Reference |
| --- | --- | --- | --- | --- |
| All-cause mortality rate  per year |  |  |  | ^[1]^ |
| Age 50-54 | 0.00299 | - | - |  |
| Age 55-59 | 0.00451 | - | - |  |
| Age 60-64 | 0.00748 | - | - |  |
| Age 65-69 | 0.01172 | - | - |  |
| Age 70-74 | 0.02026 | - | - |  |
| Age 75-79 | 0.03563 | - | - |  |
| Age 80-84 | 0.06286 | - | - |  |
| Age 85-89 | 0.10276 | - | - |  |
| Age 90-94 | 0.16165 | - | - |  |
| Age 95-99 | 0.20695 | - | - |  |
| Age 100+ | 0.23743 | - | - |  |
| HZ incidence per year |  |  |  | ^[2]^ |
| Age 50-59 | 0.00687 | 0.00293-0.01081 | Beta |  |
| Age 60-69 | 0.01106 | 0.00543-0.01670 | Beta |  |
| Age 70-79 | 0.01100 | 0.00510-0.01690 | Beta |  |
| Age 80+ | 0.01278 | 0.00496-0.02060 | Beta |  |
| Conditional probability  given HZ |  |  |  |  |
| HZ-related mortality  probability (%) per year |  |  |  | ^[3]^ |
| Age 50-59 | 0.0007 | - | - |  |
| Age 60-69 | 0.0013 | - | - |  |
| Age 70-74 | 0.0036 | - | - |  |
| Age 75-79 | 0.0075 | - | - |  |
| Age 80-84 | 0.0313 | - | - |  |
| Age 85-89 | 0.0418 | - | - |  |
| Age 90-94 | 0.1352 | - | - |  |
| Age 95-99 | 0.2651 | - | - |  |
| Age 100+ | 1.0352 | - | - |  |
| Recurrent rate (%) per year |  |  |  | ^[4]^ |
| Age 50-54 | 1.375 | - | - |  |
| Age 55-59 | 0.935 | - | - |  |
| Age 60-64 | 1.310 | - | - |  |
| Age 65-69 | 2.094 | - | - |  |
| Age 70-74 | 2.461 | - | - |  |
| Age 75-79 | 2.808 | - | - |  |
| Age 80-84 | 3.667 | - | - |  |
| Age 85+ | 2.093 | - | - |  |
| PHN probability (%) per year |  |  |  | ^[5]^ |
| Age 50-59 | 6.3 | 5.2-7.4 | Beta |  |
| Age 60-69 | 10.3 | 9.0-11.6 | Beta |  |
| Age 70-79 | 11.4 | 9.5-13.2 | Beta |  |
| Age 80+ | 30.5 | 26.4-34.6 | Beta |  |

**Table S1** (Continued)

| Variable | Base case value | Range | Distribution | Reference |
| --- | --- | --- | --- | --- |
| Other complications  probability (%) per year |  |  |  | ^[5]^ |
| Age 50-59 | 3.2 | 2.4-4.0 |  |  |
| Age 60-69 | 4.0 | 3.1-4.8 | Beta |  |
| Age 70-79 | 3.9 | 2.8-5.1 | Beta |  |
| Age 80+ | 2.6 | 1.2-4.1 | Beta |  |
| Vaccine uptake (%) | 30% | - | - | ^[6]^ |
| RZV efficacy over time |  |  |  | ^[7-11]^ |
| Initial, two doses |  |  |  |  |
| Age 50-69 | 1.000 | 0.926-1.000 | Beta |  |
| Age 70+ | 0.972 | 0.844-1.000 | Beta |  |
| Initial, one dose |  |  |  |  |
| Age 50-69 | 0.90 | 0.620-0.990 | Beta |  |
| Age 70+ | 0.69 | 0.249-0.892 | Beta |  |
| Waning rate |  |  |  |  |
| Age 50-69 | 0.027 | 0.025-0.029 | Beta |  |
| Age 70+ | 0.022 | 0.019-0.025 | Beta |  |
| ZVL efficacy over time |  |  |  | ^[12-16]^ |
| Initial |  |  |  |  |
| Age 50-59 | 0.795 | 0.734-0.843 | Beta |  |
| Age 60-69 | 0.782 | 0.721-0.830 | Beta |  |
| Age 70-79 | 0.569 | 0.514-0.619 | Beta |  |
| Age 80+ | 0.232 | 0.205-0.260 | Beta |  |
| Waning rate |  |  |  |  |
| Age 50-59 | 0.061 | 0.040-0.083 | Beta |  |
| Age 60-69 | 0.060 | 0.040-0.081 | Beta |  |
| Age 70-79 | 0.044 | 0.031-0.059 | Beta |  |
| Age 80+ | 0.019 | 0.013-0.024 | Beta |  |

**Table S2** Costs and quality-of-life inputs for the analysis [4,17-22].

| Variable | Base case value | Range | Distribution | Reference |
| --- | --- | --- | --- | --- |
| Cost of RSV | 453.55 | - | - |  |
| Cost of ZVL | 194.28 | - | - |  |
| Vaccine administration | 4.24 | - | - | ^[17]^ |
| Direct non-medical cost |  |  |  | ^[18]^ |
| Age 50-59 | 46.51 | 19.82-79.29 | Lognormal |  |
| Age 60+ | 53.64 | 28.51-92.95 | Lognormal |  |
| Direct medical cost |  |  |  | ^[4,19]^ |
| Cost of inpatient |  |  |  |  |
| Cost of PHN | 757.91 | 491.51-1143.17 | Lognormal |  |
| Cost of other complications | 543.83 | 368.66-799.53 | Lognormal |  |
| Cost with no complications | 502.05 | 335.26-744.61 | Lognormal |  |
| Cost of outpatient |  |  |  |  |
| Cost of complications | 80.79 | - | - |  |
| Cost with no complications | 67.10 | - | - |  |
| Indirect cost |  |  |  | ^[18]^ |
| Age 50-59 | 85.15 | 44.98-119.94 | Lognormal |  |
| Age 60+ | 75.42 | 27.93-163.37 | Lognormal |  |
| Per capita GDP (2023) | 12681 |  |  |  |
| Discount rate | 5% | 0-8% |  |  |
| Healthy utility adjusted |  |  |  | ^[20,21]^ |
| Age 50-59 | 0.950 | 0.949-0.951 | Beta |  |
| Age 60-69 | 0.899 | 0.897-0.901 | Beta |  |
| Age 70-79 | 0.858 | 0.855-0.861 | Beta |  |
| Age 80+ | 0.785 | 0.777-0.793 | Beta |  |
| QALY of PHN | 0.74 | 0.65-0.83 | Beta | ^[22]^ |
| QALY of other complications | 0.70 | 0.49-0.79 | Beta | ^[22]^ |
| QALY with no complications | 0.85 | 0.70-0.94 | Beta | ^[22]^ |

**Reference**

[1] China Statistics Press. China Population Census Yearbook 2020[EB/OL]. [2023-12-5]. https://www.stats.gov.cn/sj/pcsj/rkpc/7rp/zk/indexch.htm.

[2] Zhang Z, Liu X, Suo L, et al. The incidence of herpes zoster in China: A meta-analysis and evidence quality assessment[J]. Hum Vaccin Immunother, 2023, 19(2): 2228169.

[3] Teng L, Mizukami A, Ng C, et al. Cost-Effectiveness Analysis Update of the Adjuvanted Recombinant Zoster Vaccine in Japanese Older Adults[J]. Dermatol Ther (Heidelb), 2022, 12(6): 1447-1467.

[4] Sun X, Wei Z, Lin H, et al. Incidence and disease burden of herpes zoster in the population aged >/=50 years in China: Data from an integrated health care network[J]. J Infect, 2021, 82(2): 253-260.

[5] Wei J, Guiwen L, Yong X, et al. Epidemiological characteristics of herpes zoster in urban areas of Yichang city during 2016-2017 based on the Yichang Big Data Platform for Health Management[J]. Chinese Journal of Vaccines and Immunization, 2019, 25(04): 432-435.

[6] Wang Q, Yang L, Li L, et al. Willingness to Vaccinate Against Herpes Zoster and Its Associated Factors Across WHO Regions: Global Systematic Review and Meta-Analysis[J]. JMIR Public Health Surveill, 2023, 9: e43893.

[7] Cunningham A L, Lal H, Kovac M, et al. Efficacy of the Herpes Zoster Subunit Vaccine in Adults 70 Years of Age or Older[J]. N Engl J Med, 2016, 375(11): 1019-32.

[8] Lal H, Cunningham A L, Godeaux O, et al. Efficacy of an adjuvanted herpes zoster subunit vaccine in older adults[J]. N Engl J Med, 2015, 372(22): 2087-96.

[9] Strezova A, Diez-Domingo J, Al Shawafi K, et al. Long-term Protection Against Herpes Zoster by the Adjuvanted Recombinant Zoster Vaccine: Interim Efficacy, Immunogenicity, and Safety Results up to 10 Years After Initial Vaccination[J]. Open Forum Infect Dis, 2022, 9(10): ofac485.

[10] Boutry C, Hastie A, Diez-Domingo J, et al. The Adjuvanted Recombinant Zoster Vaccine Confers Long-Term Protection Against Herpes Zoster: Interim Results of an Extension Study of the Pivotal Phase 3 Clinical Trials ZOE-50 and ZOE-70[J]. Clin Infect Dis, 2022, 74(8): 1459-1467.

[11] Prosser L A, Harpaz R, Rose A M, et al. A Cost-Effectiveness Analysis of Vaccination for Prevention of Herpes Zoster and Related Complications: Input for National Recommendations[J]. Ann Intern Med, 2019, 170(6): 380-388.

[12] Morrison V A, Johnson G R, Schmader K E, et al. Long-term persistence of zoster vaccine efficacy[J]. Clin Infect Dis, 2015, 60(6): 900-9.

[13] Schmader K E, Oxman M N, Levin M J, et al. Persistence of the efficacy of zoster vaccine in the shingles prevention study and the short-term persistence substudy[J]. Clin Infect Dis, 2012, 55(10): 1320-8.

[14] Oxman M N, Levin M J, Johnson G R, et al. A vaccine to prevent herpes zoster and postherpetic neuralgia in older adults[J]. N Engl J Med, 2005, 352(22): 2271-84.

[15] Tseng H F, Harpaz R, Luo Y, et al. Declining Effectiveness of Herpes Zoster Vaccine in Adults Aged >/=60 Years[J]. J Infect Dis, 2016, 213(12): 1872-5.

[16] Baxter R, Bartlett J, Fireman B, et al. Long-Term Effectiveness of the Live Zoster Vaccine in Preventing Shingles: A Cohort Study[J]. Am J Epidemiol, 2018, 187(1): 161-169.

[17] Xixi Z, Li L, Lijun L, et al. Costs of the Expanded Program on Immunization in county-level centers for disease control and prevention and vaccination units in several areas of China: a cross-sectional survey in 2021[J]. Chinese Journal of Vaccines and Immunization, 2023, 29(02): 131-137.

[18] Fei W, Peng-Fei J, Fan-Yue M, et al. Economic burden and health-related quality of life of herpes zoster disease in rural areas of Jiang-su Province[J]. Chinese Journal of Disease Control and Prevention, 2022, 26(06): 736-739.

[19] Chen P, Chen Z, Xiao Y, et al. Characteristics and economic burden of hospitalized patients with herpes zoster in China, before vaccination[J]. Hum Vaccin Immunother, 2023, 19(3): 2268990.

[20] Zhang J, Xu L, Li J, et al. Gender differences in the association between body mass index and health-related quality of life among adults:a cross-sectional study in Shandong, China[J]. BMC Public Health, 2019, 19(1): 1021.

[21] Lei P, Xu L, Nwaru B I, et al. Social networks and health-related quality of life among Chinese old adults in urban areas: results from 4th National Household Health Survey[J]. Public Health, 2016, 131: 27-39.

[22] Zhang Q, Huang Z S, Hu Q Q, et al. Quality of life and risk factors in patients with herpes zoster[J]. Zhonghua Yi Xue Za Zhi, 2022, 102(42): 3395-3400.
